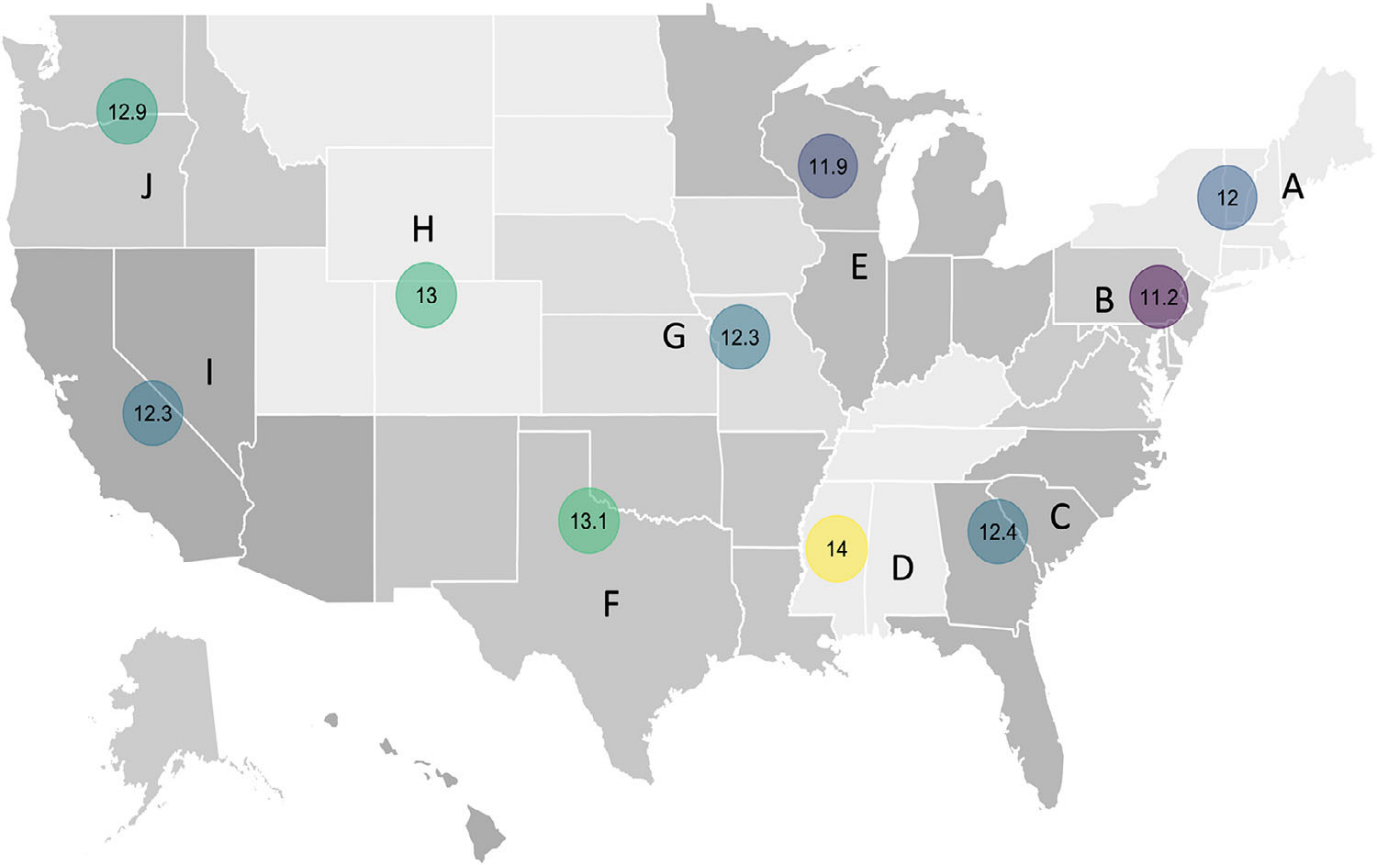# Regional Difference in Dementia Incidence in Older Adults

**DOI:** 10.1002/alz.091877

**Published:** 2025-01-09

**Authors:** Christina S. Dintica, Amber L Bahorik, Feng Xia, W John Boscardin, Kristine Yaffe

**Affiliations:** ^1^ University of California, San Francisco, San Francisco, CA USA; ^2^ NCIRE‐The Veterans Health Research Institute, San Francisco, CA USA; ^3^ University of California San Francisco / San Francisco VA Medical Center, San Francisco, CA USA

## Abstract

**Background:**

Studies in prevalence and incidence in the United States have been limited to clinical populations and single site studies, therefore, there is a notable lack in estimates of regional differences in dementia incidence and the drivers of such disparities.

**Methods:**

We included 1,268,599 US Veterans Health Administration (VHA) dementia‐free patients aged 65 years or older living within the U.S. with a residential zip code from year 2000‐2021. Dementia incidence was estimated according to US geographical areas from residential zip codes, based on the 10 Centers for Disease Control and Prevention (CDC) National Center for Chronic Disease Prevention and Health Promotion (NCCDPHP) regions, labeled from A to J. Incident diagnosis of dementia was ascertained using the 9th and 10th editions of the International Classification of Diseases. Poisson regression models adjusted for age were used to asses differences in dementia incidence based on geographical regions, and the sensitivity of the findings were evaluated by accounting for competing risk of death.

**Results:**

Among the 1,268,599 study participants (mean age 73.9 [SD, 6.1] years; 25 335 women [2%]; 15.5% received a diagnosis of dementia over a mean follow‐up of 12.6 years. Unadjusted incidence of dementia per 1000 person‐years was the lowest for the Northeast region, B and the highest in in the Southeastern region D (Figure 1). Residence within the Southeastern region D was associated a 27% higher risk of dementia, 19% higher risk in the Southern region F, 17% the midwestern region H and Northwestern region J, 12% for Southeastern region C; the remaining regions had an increased risk <10% compared to region B (Table 1). Additional adjustment for sex and race, and accounting for the competing risk of death produced similar results.

**Conclusion:**

Among older adults who received care at VHA medical centers, there were significant geographical differences in dementia incidence across the U.S, suggesting important roles for geographically patterned risk factors. Identifying geographical differences in dementia incidence allows for a more strategic and targeted approach to healthcare planning, public health interventions, and policy development.